# Do ambient urban odors evoke basic emotions?

**DOI:** 10.3389/fpsyg.2014.00340

**Published:** 2014-04-23

**Authors:** Sandra T. Glass, Elisabeth Lingg, Eva Heuberger

**Affiliations:** ^1^Division of Clinical Pharmacy and Diagnostics, Department of Pharmaceutical Chemistry, University of ViennaVienna, Austria; ^2^Department of Health Sciences and Technology, Institute for Human Movement Sciences and Sport, ETH ZurichZurich, Switzerland; ^3^Division of Clinical Psychology and Psychotherapy, Department of Psychology, Saarland UniversitySaarbruecken, Germany; ^4^Pharmaceutical Biology, Department of Pharmacy, Saarland UniversitySaarbruecken, Germany

**Keywords:** city odors, basic emotions, autonomic nervous system, hedonic valence, odor intensity

## Abstract

Fragrances, such as plant odors, have been shown to evoke autonomic response patterns associated with Ekman's (Ekman et al., 1983) basic emotions happiness, surprise, anger, fear, sadness, and disgust. Inducing positive emotions by odors in highly frequented public spaces could serve to improve the quality of life in urban environments. Thus, the present study evaluated the potency of ambient odors connoted with an urban environment to evoke basic emotions on an autonomic and cognitive response level. Synthetic mixtures representing the odors of disinfectant, candles/bees wax, summer air, burnt smell, vomit and musty smell as well as odorless water as a control were presented five times in random order to 30 healthy, non-smoking human subjects with intact sense of smell. Skin temperature, skin conductance, breathing rate, forearm muscle activity, blink rate, and heart rate were recorded simultaneously. Subjects rated the odors in terms of pleasantness, intensity and familiarity and gave verbal labels to each odor as well as cognitive associations with the basic emotions. The results showed that the amplitude of the skin conductance response (SCR) varied as a function of odor presentation. Burnt smell and vomit elicited significantly higher electrodermal responses than summer air. Also, a negative correlation was revealed between the amplitude of the SCR and hedonic odor valence indicating that the magnitude of the electrodermal response increased with odor unpleasantness. The analysis of the cognitive associations between odors and basic emotions showed that candles/bees wax and summer air were specifically associated with happiness whereas burnt smell and vomit were uniquely associated with disgust. Our findings suggest that city odors may evoke specific cognitive associations of basic emotions and that autonomic activity elicited by such odors is related to odor hedonics.

## Introduction

In urban environments both residents and visitors are surrounded by a multitude of odors which, along with visual, acoustic and haptic sensations, accompany and shape their individual perceptual experiences. These contextual stimuli are believed to be encoded in episodic memory along with an event and with the emotions experienced at that event and can serve as triggers for the retrieval of event details, such as the experienced emotions, on subsequent encounters (Jellinek, 1997; Chu and Downes, 2000). A number of laboratory studies have shown that highly emotional stimuli are more efficient triggers of episodic memory than emotionally neutral ones (Koenig and Mecklinger, 2008) and that odors are such highly emotional cues (Chu and Downes, 2002; Goddard et al., 2005; Willander and Larsson, 2007). Particularly in big cities the olfactory environment might have great impact on the experience of public spaces of both inhabitants and visitors. For instance, feelings of pleasure might be experienced in the vicinity of a bakery emitting the smell of freshly baked bread or in a public garden with fragrant flowers (Weber and Heuberger, 2008). By contrast, negative emotions might be elicited in places where people crowd together in confined spaces, such as public transport, or in other places that are experienced as constricted, smelly, and unpleasant. To counteract such possible negative experiences efforts are being made to increase the pleasantness of the urban olfactory environment (Hosey, 2013). Although inducing positive emotions in highly frequented public spaces could be a simple and efficient means to improve the quality of life in urban environments no research exists to date that addresses this question.

One way to assess the potency of sensory stimuli to induce affective reactions is to measure self-reported emotions together with associated changes in autonomic nervous system (ANS) activity. Although the debate is still ongoing as to whether or not emotion-specific autonomic activity exists (see Kreibig, 2010 for an up-to-date review) and many studies have failed to reveal such specificity (Aue and Scherer, 2008), a considerable number of reports exists in favor of the hypothesis of emotion-specific physiological activity (Friedman, 2010; Stephens et al., 2010). The issue of specific physiological patterns is intrinsically linked with the concept of basic emotions, i.e., a limited number of primary affective states which are generated universally and prototypically in response to environmental demands and may be regarded as discrete points in dimensional affective space (Christie and Friedman, 2004). The discussion about unique autonomic response patterns allowing to distinguish between these basic emotions has received great support by the studies of Ekman et al. (1983) in which six basic emotions, i.e., happiness, surprise, anger, fear, sadness and disgust, were evoked by generating directed emotion-prototypical facial expressions, and by reliving an emotional experience. The authors reported that they were able to differentiate between positive and negative emotions as well as among negative emotions based on a decision tree that took into account changes in heart rate and skin temperature. More recent investigations have demonstrated that viewing these emotion-prototypic facial expressions (Collet et al., 1997) as well as viewing emotional film clips and listening to emotional music (Christie and Friedman, 2004; Etzel et al., 2006) may induce emotion specific autonomic response patterns. Also, stimuli from the gustatory domain (Rousmans et al., 2000) have been found to induce emotional states with distinguishable autonomic patterns.

In regard to olfaction, several investigations have revealed emotion-specific ANS response patterns (Alaoui-Ismaili et al., 1997a,b; Robin et al., 1999; Vernet-Maury et al., 1999; Bensafi et al., 2002b; Moller and Dijksterhuis, 2003). However, comparisons between verbal reports and physiological activity of the elicited emotions often showed a mismatch between these two response systems and the valence of the odor evoked affective reaction seems to be associated with the hedonic valence of the odor (Brauchli et al., 1995; Bensafi et al., 2002a; Delplanque et al., 2008; Weber and Heuberger, 2008). Alaoui-Ismaili et al. (1997a) were able to link both verbal responses and psychophysiological correlates of Ekman's basic emotions to a number of odors that differed in hedonic quality. In this study, they presented vanillin and menthol which were rated as pleasant and methyl methacrylate and propionic acid which were rated as unpleasant to 44 healthy students and recorded several electrodermal and cardio-respiratory parameters. In addition, subjects had to indicate which of the six basic emotions was evoked by each of these odors. The authors reported that the pleasant odors evoked happiness and surprise regarding both verbal reports and autonomic response patterns. The unpleasant odors, however, evoked disgust as the verbal response but anger as the autonomic response. Another study by the same group with a different set of odorants confirmed the association between the hedonic valence of the odors and the emotion specific autonomic response patterns (Alaoui-Ismaili et al., 1997b). In regard to the relationship between hedonic odor rating and the valence of the evoked emotion, an interesting finding was reported by Robin et al. (1999). Based on the observation that eugenol is contained in many materials used in restorative dental treatments (Sarrami et al., 2002), these authors compared basic emotions elicited by eugenol odor in fearful and non-fearful dental care subjects and reported that such prior experience with the odor modulated both the hedonic evaluation of the odor and the emotional response, i.e., in non-fearful subjects eugenol odor was rated as pleasant and evoked positive emotions, i.e., happiness and surprise, while in fearful participants the odor was rated as unpleasant and evoked negative emotions, i.e., fear, anger, and disgust.

In regard to verbal reports of odor induced affective reactions, a study by Bensafi et al. (2002b) in 12 healthy participants with 12 different food odors ranging from very pleasant to very unpleasant showed that from seven emotional terms “joy” and “disgust” were chosen more often than the other emotion terms. In addition, facial EMG activity differentiated between these two emotions. An explanation for these findings can be found in the results of Chrea et al. (2009) who argued that the small number of basic emotions may be insufficient and inappropriate to describe the multitude of emotional states which can be elicited by olfactory stimuli and that olfaction-specific dimensions were better suited to account for verbal descriptions of odor induced feelings (Delplanque et al., 2012). These authors presented a 6 to 7-factorial model that describes the semantic space of affective verbal responses to odors and showed that four of these dimensions which were related to disgust, happiness/well-being, sensuality/desire, and energy were shared by different cultures (Ferdenzi et al., 2011, 2013).

Concentrating on verbal reports of basic emotions triggered by olfactory cues Croy et al. (2011) took a different approach and came to slightly different conclusions. Instead of presenting preselected odors, they interviewed 119 healthy subjects about free associations between odors and each of the six basic emotions. As a control they asked another 97 participants about their associations of the basic emotions with pictures. The results of this study showed that the vast majority of subjects were able to report an odor that elicited happiness or disgust. Olfactory cues associated with anxiety were reported by 75% of the participants. In contrast, only 50% of the subjects were able to identify an olfactory elicitor for sadness and anger (Croy et al., 2011). The authors concluded that only a limited number of emotions, i.e., happiness, anxiety, and disgust, can be elicited verbally by olfactory cues.

The present study aimed to evaluate whether affective responses are evoked by ambient odors connoted with the City of Vienna. Specifically, we tested whether such odors elicit emotion specific autonomic response patterns and verbal associations with the basic emotions. Notwithstanding the above mentioned findings on the olfactory semantic space (Ferdenzi et al., 2011, 2013) we favored a discrete (basic) emotions model over a two-dimensional (valence-by-arousal) approach as a theoretical framework for our study. According to Levenson (2003) the former allows for more finely tuned responses than the latter not only at the physiological but also at the endocrine, cognitive, and behavioral level. In our view, this constitutes a functional advantage in the case of olfactory triggered emotions. For example, consider disgust and fear. Both emotions possess high negative valence, are highly arousing, and are associated with withdrawal behavior (Christie and Friedman, 2004). However, while disgust is associated with objects that are potentially harmful after ingestion (such as spoiled food because it may be toxic), or direct skin contact (such as excrement because it may carry germs), fearful stimuli, such as fire or an aggressor, are threatening because they may inflict severe injuries. Thus, one could generalize that disgusting stimuli convey a threat to the body interior while fearful stimuli impose a threat to the outside of the body. In regard to the responses, disgust eliciting stimuli require bodily reactions that help to remove the threat from the organism, such as vomiting (Croy et al., 2013). Fearful stimuli, on the other hand, should initiate behavior that mobilizes enough energy to remove oneself from the source of danger. Responding in the one or the other way of course requires a completely different sort of preparation, also in the ANS (Levenson, 2003). Olfaction is a proximal sense, and once an odor can be perceived its source is quite close. Consequently, the appropriate response, disgust and regurgitation or fear and flight in this example, must be induced quickly. Therefore, in response to odors we think that unique physiological patterns as predicted by the basic emotions model have greater adaptive value than adopting mere approach-avoidance behavior as the dimensional model would predict.

In order to increase the emotional valence of the odorous stimuli we combined the experimental approach reported by Croy et al. (2011) with that of other studies, i.e., rather than selecting odors on a random basis we first conducted semi-structured interviews in a larger sample of Viennese residents (*N* = 50) (Weber and Heuberger, 2011). Specifically, we asked them to think of and narrate to the experimenter an experience in the City of Vienna which involved one of the six basic emotions. Subjects were free to decide in how much detail they wanted to describe the experience. Then, they had to name at least one odor that was associated with this memory. In addition, the participants rated how emotional and how vivid the memory was, how brought back in time they felt when they thought of the odor, and how specific the odor was for the memory. For each basic emotion the same questions were asked. To identify olfactory stimuli that were specific for a given basic emotion the count of each nominated odor was assessed for each basic emotion. While in this study odor associated memories were reported for each of the basic emotions, the interviews demonstrated that only a very small number of the reported odors were specific for a particular basic emotion, such as “vomit” for disgust. Thus, to obtain the (potentially) full range of odor evoked basic emotions we decided to select odors that were specific in regard to their emotional impact even though they were reported by only a small number of participants in the preceding interviews. The next task in the stimulus selection process consisted of “translating” the odor names into “perfumes” that involved a manageable number of chemical constituents but would still be clearly recognizable by the tested sample. Thus, we identified the character impact compounds of the selected odors and created synthetic mixtures that best represented their olfactory properties. We limited the number of constituents to three. Several challenges had to be met during this step. For instance, in the case of “burnt smell” and “candles” a character impact compound (prop-2-enal, also known as acrolein) could not be used due to its toxicity. To circumvent this issue, we decided to use other non-toxic chemicals with appropriate olfactory properties (see Table 1). In the case of “summer air” the search for suitable character impact compounds did not yield satisfactory results due to the ambiguity of the odor concept so that we decided to choose a green note reminiscent of leaves and grass. We considered this to be the best choice because one of the most frequented places in Vienna during summer time is the so-called “Donauinsel,” a man-made island at the Danube River that is vegetated with meadows and trees. Ultimately, we were interested in the question whether the emotional valence of the selected odors would transfer to another sample of subjects, i.e., whether the chosen odor representations would elicit the same basic emotions in a different sample of subjects. In our view this would constitute a basic prerequisite for the creation of olfactory environments which elicit distinct emotional states.

**Table 1:** **Chemical composition, concentration of constituents, and association with the basic emotions for all olfactory stimuli**.

**Odor**	**Components**	**Basic emotion**
Summer air	Leaf alcohol (Z-hex-3-en-1-ol) (0.1% v/v in PG)	Happiness
Candles (bees wax)	Methyl 2-phenylacetate (0.5% v/v in PG)	Surprise
Disinfectant	Isopropyl alcohol (propan-2-ol) (50% v/v in PG)	Fear
Burnt smell	Guaiacol (2-methoxyphenol) (10% v/v in PG)	Anger
Musty smell	(±)-geosmin [(4R,4aR,8aS)-4,8a-dimethyl-1,2,3,4,5,6,7,8-octahydronaphthalen-4a-ol] (0.01% m/V in MeOH/EtOH 96%, 9 pt), green pea pyrazine [2-methoxy-3,5 or 6-(propan-2-yl)pyrazine] (0.1% v/v in EtOH 96%, 1 pt)	Sadness
Vomit	Butanoic acid (10% v/v in PG, 2 pt), isovaleric acid (3-methylbutanoic acid) (10% v/v in PG, 7 pt), hydrochloric acid (36% v/v, 1 pt)	Disgust

*EtOH, ethanol; MeOH, methanol; PG, propylene glycol (propane-1,2-diol); m/v, mass per volume; v/v, volume per volume; pt, parts*.

## Materials and methods

### Ethics statement

The study was performed in accordance with the Declaration of Helsinki on Biomedical Research Involving Human Subjects and with the guidelines of the Institutional Review Board at the University of Vienna. All participants provided written informed consent, received financial compensation for their time commitment, and were free to withdraw from the study at any time.

### Olfactory screening

In a first step, the olfactory acuity of the subjects who enrolled for the study was determined using the odor discrimination and identification tests from the Sniffing Sticks olfactory test battery (Hummel et al., 1997). The discrimination test consisted of odor triplets, of which two fragrances were identical distractors and one was the target that smelled different from the distractors. Each subject was required to identify the target odor. The criterion for inclusion in the subsequent psychophysiological study was the correct identification of 11 (out of 16) triplets. In the odor identification test, each subject had to sample a target odor and pick the correct odor name among four written alternatives. The criterion for inclusion in the subsequent psychophysiological study was the correct identification of at least 13 (out of 16) odors. Only participants who successfully identified and discriminated the presented odors were tested in the main study, i.e., the psychophysiological measurements, which was conducted on a different day than the olfactory screening.

### Subjects

In total, 30 healthy and neurologically inconspicuous individuals (15 males) between the age of 18 and 34 (mean age 24 ± 4 years) participated in the main study. All participants had normal blood pressure, no history of olfactory deficits, allergies to fragrances, or neurological diseases. None of the women were pregnant and all participants were non-smokers. All subjects were Viennese residents and recruited by advertisement at the University of Vienna. They received financial compensation for their time commitment.

### Olfactory stimuli

The olfactory stimuli used in the main study were synthetic mixtures representing the odors of warm summer air, candles/bees wax, disinfectant, burnt smell, musty smell, and vomit. The number of components in each mixture was limited to three. Odorless water was used as a control. For each odor, the chemical composition and association with the basic emotions is given in Table 1.

### Experimental design and procedures

The psychophysiological study took place in a temperature controlled and well ventilated room at the Department of Clinical Pharmacy and Diagnostics at the University of Vienna. The participants were seated in a comfortable chair and their non-dominant hand was placed on a soft pillow.

Skin conductance, forearm muscle activity, eye-blink rate, skin temperature, as well as breathing and heart rate were measured simultaneously and in real-time via MP100WSW hardware (Biopac Systems, Inc., Santa Barbara, California, USA) and AcqKnowledge® software (V 3.9.0.17, ^©^ 1992–2007, BIOPAC Systems, Inc., Santa Barbara, California, USA) with a sampling rate of 1000 Hz. All signals were filtered by means of hardware-based filters included in the amplifiers. Skin conductance was recorded using a GSR100B amplifier and 6 mm inner diameter Ag/AgCl finger electrodes (TSD203) via the constant voltage (0.5 V) technique. Electrodes were filled with conductive gel and placed on the second phalanx of the middle and the index finger of the non-dominant hand with non-caustic adhesive tape. Electrode positioning was in compliance with traditional recommendations (Fowles et al., 1981). The signal was low pass filtered at 1 Hz. Surface electromyogram (EMG) was recorded with a EMG100B amplifier, Ag/AgCl surface electrodes (EL208S), and adhesive disks (ADD208). Electromyographic activity was recorded by placing two electrodes, which were filled with conductive gel, over the forearm flexors of the non-dominant hand as suggested by Cacioppo et al. (1990). The raw EMG signal was band pass filtered (1–500 Hz), with a notch filter centered at 50 Hz, and converted to an average root-mean-square (rms) signal (time constant 500 ms, baseline removal). Eye-blinks were recorded by means of a EOG100B amplifier, Ag/AgCl surface electrodes (EL208S), and adhesive disks (ADD204). Two electrodes, which were filled with conductive gel, were placed over the left orbicularis oculi muscle on a vertical line (Stern et al., 2001). The signal was low pass filtered at 35 Hz and a 50 Hz notch filter was employed. ST was measured using a SKT100B amplifier and a fast response thermistor (TSD202A). The sensor was placed on the middle of the back of the non-dominant hand with non-caustic adhesive tape. The signal was low pass filtered at 1 Hz. Heart rate was measured via a ECG100C amplifier and Ag/AgCl surface electrodes (Skintact®, T601, Leonard Lang GmbH, Austria). The ECG signal was band pass filtered (0.05–35 Hz), with a 50 Hz notch filter. Heart rate was detected from the ECG via an integrated rate detector (peak interval window 40–180 bpm, noise rejection 5% of peak) and sampled at 250 Hz. Breathing was recorded via a RSP100C amplifier and a breathing belt (BIOPACTSD201) with an integrated electrical sensor. The belt was placed below the sternum and above the ECG electrodes. Any change in the belt's length was recorded by the electric sensor. The signal was low pass filtered at 10 Hz.

To each subject, the six olfactory stimuli and odorless water as a control stimulus were presented on sniffing stripes (Primavera Life GmbH, Germany) by one of two experimenters. 5 ml of each liquid stimulus were filled into 20 ml screw-cap brown glass vials coded by a number from 1 to 7. Stimulus concentration was kept constant by dipping the sniffing stripe into the vial until it reached the ground. To prevent the adulteration of the experimental stimuli with odors stemming from the hand of the experimenter, the experimenter wore cotton gloves. The stimuli were presented in randomized order. Each stimulus was presented 5 times. Stimulus presentation was synchronized with inspiration via the observation of the respiration channel and was marked in the recording by means of a hand switch. At the onset of inspiration the experimenter held a sniffing stripe soaked with the appropriate stimulus approximately 2 cm under the nostrils of the subject. Each stimulus presentation lasted for one breathing cycle. Subjects were instructed to breathe normally whether or not a stimulus was presented. The interstimulus interval was 2 min. Each stripe was used only once and discarded into a sealed container after use. The average duration of the experiment was 80 min. There was a 10 min baseline phase before the first odor presentation to ensure that all ANS parameters returned to their baseline levels before the first odor presentation took place.

After the psychophysiological measurements were finished, all participants completed a set of different questions. They had the opportunity to smell each of the odors again before giving their answers to the questions. The participants were asked to produce a verbal label for each odor. Using Likert scales they were then required to indicate the strength (1 = “very weak” and 10 = “very strong”) of the association with each of the six basic emotions (i.e., happiness, surprise, anger, fear, sadness, and disgust). For a given odor, the emotion that received the highest rating was given one point, whereas all other emotions received zero points. If for a given odor two or more emotions received equal ratings, then one point was assigned to the category “no or unspecific association.” Likert scales were also used to acquire data about the intensity of the odors (1 = “very weak” and 10 = “very strong”), the valence of the odors (1 = “very unpleasant” and 10 = “very pleasant”) and the familiarity of the odors (1 = “very unfamiliar” and 10 = “very familiar”).

### Data analysis

All recordings were edited offline for movement, breathing or electronic artefacts. No additional offline filtering was applied to the data. Since emotional reactions are quickly unfolding phasic events, a time window of 10 s post-stimulus was chosen (Ekman, 1992). The mean for each parameter was calculated across trials for each of the seven odor conditions. Only the first four blocks were included in the data analysis, since the participants showed signs of fatigue in the last (fifth) block due to the overall length of the experiment. Changes in muscle tension (rms EMG), number of eye-blinks, ST, number of breaths and heart rate were expressed as the difference between the respective mean of the prestimulus (10 s) and the post-stimulus (10 s) time interval. The change in heart rate variability (HRV) was calculated as the difference between the standard deviation (SD) of the heart rate before (10 s) and after (10 s) stimulus onset. The amplitude as well as the latency and the recovery time of the skin conductance response (SCR) were analyzed separately. The time window for the latency response was 1–4 s after stimulus onset. The criterion for a SCR to be included in the analysis was 0.05 μS/cm^2^ (Boucsein, 1988). In order to be able to compare the SCR amplitudes (SCR-a) across subjects, each amplitude value in a given odor condition was divided by the corresponding maximum value across all trials (Schandry, 1989).

### Statistical analysis

To evaluate the impact of the different odor stimuli One-Way repeated measures ANOVAs with the within-subjects factor “odor” were conducted for each of the psychophysiological parameters and for each of the odor ratings (i.e., intensity, valence, and familiarity). Degrees of freedom were adjusted via the Greenhouse-Geisser method. *Post-hoc* pairwise comparisons were calculated using Bonferroni corrected *P*-values to control for alpha inflation. Two-sided Pearson product-moment correlations were calculated to identify potential relationships between the ANS parameters and the different odor ratings as well as between the odor ratings themselves. These analyses were conducted with the data of 16 subjects. For 14 subjects the data was not sufficient (in most cases due to SCRs that did not meet the amplitude or temporal criteria) to allow for further analyses.

The association between each odor and the six basic emotions was analyzed using a Pearson's χ^2^ test (*N* = 30). The observed associations were compared to hypothetical associations based on our previous findings (Weber and Heuberger, 2011).

## Results

### Autonomic nervous system parameters

**Figure 1:**
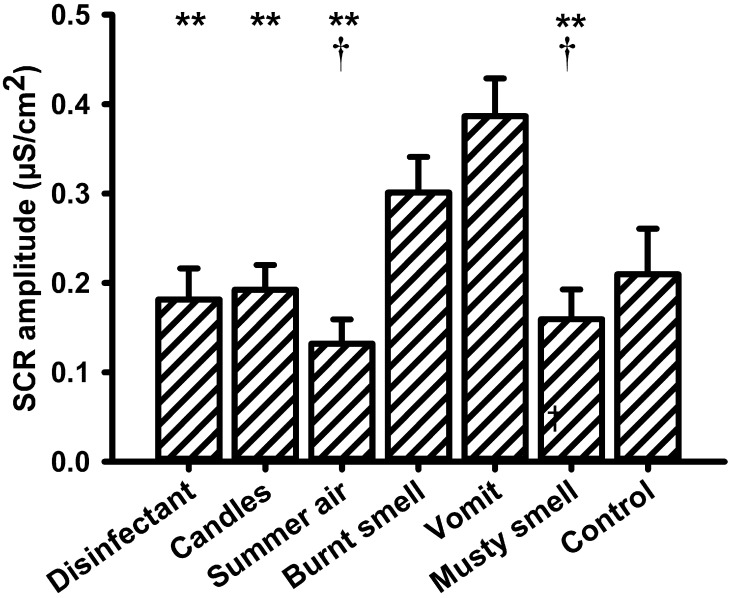
**Mean values (and s.e.m.) of the amplitude of the skin conductance response (SCR) to all olfactory stimuli**. ^**^Differs significantly (*P* < 0.005) from “vomit,” ^†^differs significantly (*P* < 0.05) from “burnt smell.”

**Figure 2:**
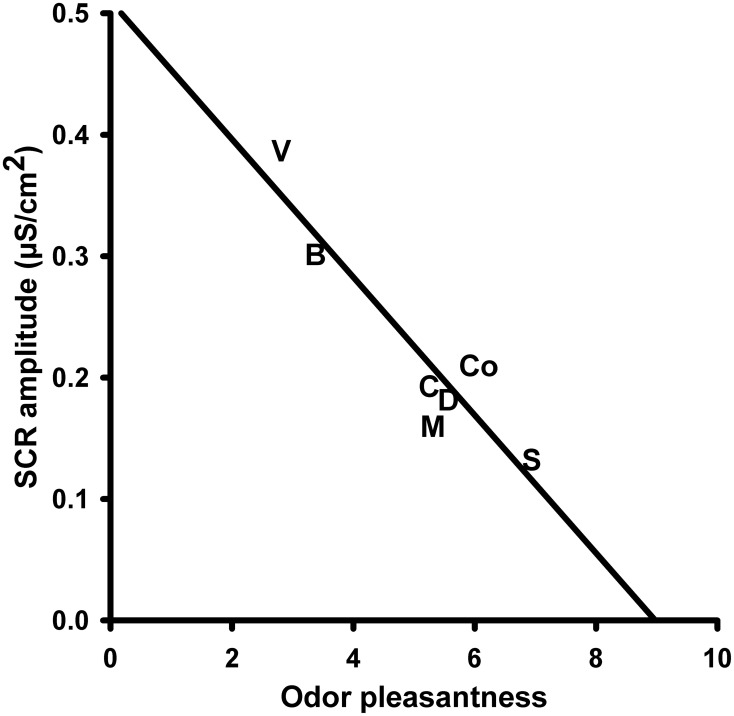
**Correlation between the amplitude of the skin conductance response (SCR) to the olfactory stimuli and perceived odor pleasantness**. S, Summer air; C, Candles; D, Disinfectant; B, Burnt smell; M, Musty smell; V, Vomit; Co, Control.

The *amplitude of the skin conductance responses* (SCR-a) varied significantly with the presented olfactory stimulus. A One-Way repeated measures ANOVA with the within-subjects factor “odor” revealed a significant main effect for the factor “odor” [*F*_(6, 90)_ = 7.579, *P* = 0.000]. Mean values of SCR-a are depicted in Figure 1. *Post-hoc* pairwise comparisons showed that the unpleasant odor “vomit” elicited significantly larger responses than all other odors (“disinfectant”: *P* = 0.002, “candles”: *P* = 0.004, “summer air”: *P* = 0.001, and “musty smell”: *P* = 0.002) except “burnt smell” and odorless water (i.e., the control stimulus). We also found a significant difference between “summer air” and “burnt smell” (*P* = 0.022) and between “burnt smell and “musty smell” (*P* = 0.031). In addition, there was a significant negative correlation between SCR-a and the odor valence ratings (*N* = 7, *r* = −0.927, *P* = 0.003; Figure 2), i.e., the amplitude of the SCR decreased with the perceived pleasantness of a fragrance. This correlation was unaffected by either intensity (*N* = 7, *r* = −0.962, *P* = 0.002) or familiarity ratings (*N* = 7, *r* = −0.951, *P* = 0.003) as revealed by partial correlation analyses. The correlation between SCR-a and perceived intensity was marginally significant only after controlling for the perceived pleasantness of the odors (*N* = 7, *r* = −0.768, *P* = 0.074) and significant after controlling for ratings of familiarity (*N* = 7, *r* = −0.823, *P* = 0.044) indicating that SCR-a increased with the perceived intensity of an odor. There was no significant correlation between SCR-a and familiarity (*P* > 0.1).

The *latency* and *the half recovery time of the skin conductance response* were analyzed using One-Way repeated measures ANOVAs with the within-subjects factor “odor” but no significant main effects were found (all *P* > 0.1; mean values and s.e.m. of the latency and half recovery time of the SCR are given in Table S1 in the supplementary material). Neither the correlation analyses between the subjective odor ratings (i.e., perceived odor pleasantness, intensity, and familiarity) and the latency of the SCR nor those between the subjective odor ratings and the half recovery time of the SCR revealed any significant relationships (all *P* > 0.1).

**Figure 3:**
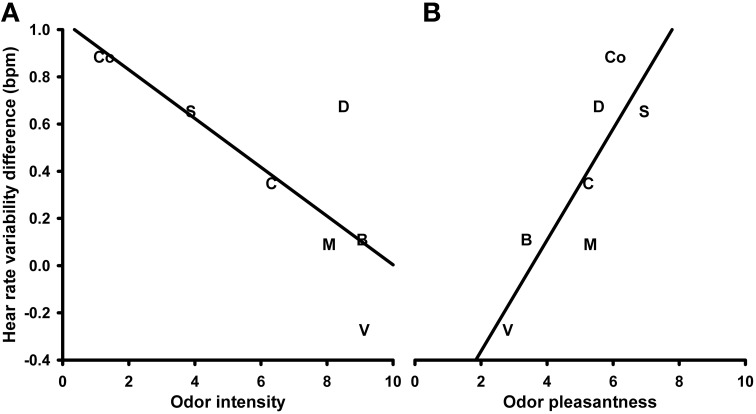
**Correlation between heart rate variability in response to the olfactory stimuli and perceived odor intensity (A) and pleasantness (B), respectively**. S, Summer air; C, Candles; D, Disinfectant; B, Burnt smell; M, Musty smell; V, Vomit; Co, Control.

Changes in *heart rate variability (HRV)* in response to the different olfactory stimuli were analyzed using a One-Way repeated measures ANOVA with the within-subjects factor “odor.” The analysis revealed no significant effects (*P* > 0.1; mean values and s.e.m. of the HRV changes are given in Table S1 in the supplementary material). The correlation analysis, however, showed a significant negative correlation between HRV changes and the odor intensity ratings (*N* = 7, *r* = −0.763, *P* = 0.046; Figure 3A). This correlation remained significant after controlling for the ratings of familiarity (*N* = 7, *r* = −0.905, *P* = 0.013) but disappeared after controlling for their perceived pleasantness (*P* > 0.1). The correlation between HRV changes and the odor valence ratings was also significant (*N* = 7, *r* = 0.843, *P* = 0.017; Figure 3B). This correlation remained significant after controlling for the perceived familiarity of the odors (*N* = 7, *r* = 0.846, *P* = 0.034), but disappeared after controlling for their perceived intensity (*P* > 0.1). The correlation between HRV changes and the familiarity ratings revealed no significant relationship (*P* > 0.1).

**Figure 4:**
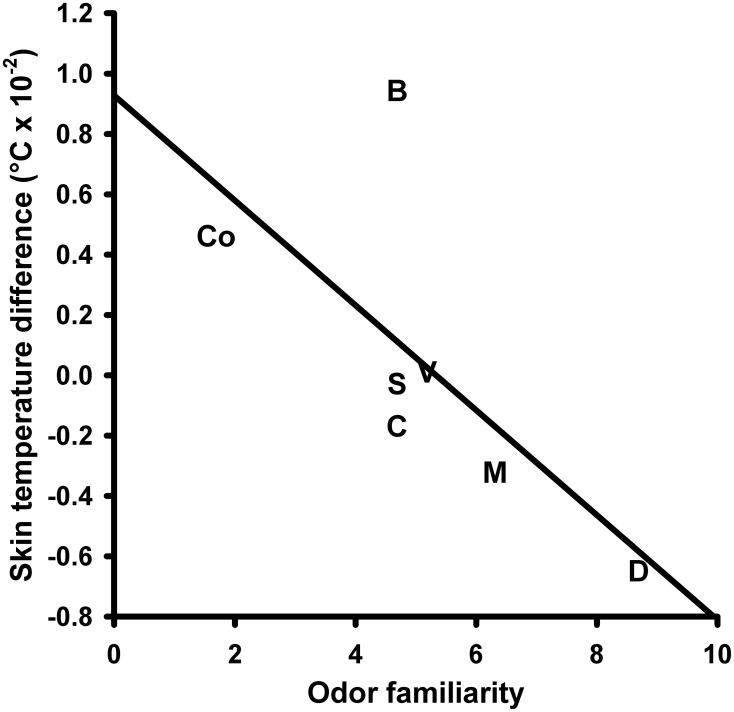
**Correlation between the skin temperature response to the olfactory stimuli and perceived odor familiarity**. S, Summer air; C, Candles; D, Disinfectant; B, Burnt smell; M, Musty smell; V, Vomit; Co, Control.

*Skin temperature* (ST) responses to the olfactory stimuli did not change depending on the different olfactory stimuli. A One-Way repeated measures ANOVA with the within-subjects factor “odor” did not yield a significant main effect for this factor [*F*_(6, 90)_ = 2.664, *P* = 0.068; mean values and s.e.m. of the ST changes are given in Table S1 in the supplementary material]. A significant negative correlation was revealed between the ST responses and the odor familiarity ratings (*N* = 7, *r* = −0.697, *P* = 0.041; Figure 4).

*Number of breaths*, *heart rate*, *number of eye-blinks* and *forearm muscle activity* did not vary dependent on the presented olfactory stimuli. Neither the One-Way repeated measures ANOVAs with the within-subjects factor “odor” nor the correlation analyses (with the valence, intensity, and familiarity ratings) revealed a significant result (all *P* > 0.1; mean values and s.e.m. of the changes of number of breaths, heart rate, number of eye-blinks, and forearm muscle activity are given in Table S1 in the supplementary material).

### Valence, intensity, and familiarity ratings

**Figure 5:**
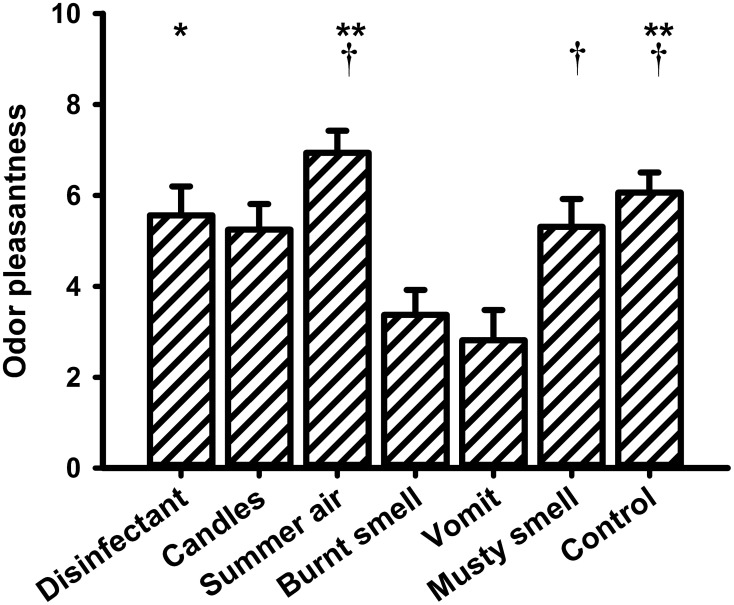
**Mean values (and s.e.m.) of the ratings of odor valence**. ^**^Differs significantly (*P* < 0.01) from “vomit,” ^*^differs significantly (*P* < 0.05) from “vomit,” ^†^differs significantly (*P* < 0.05) from “burnt smell.”

**Figure 6:**
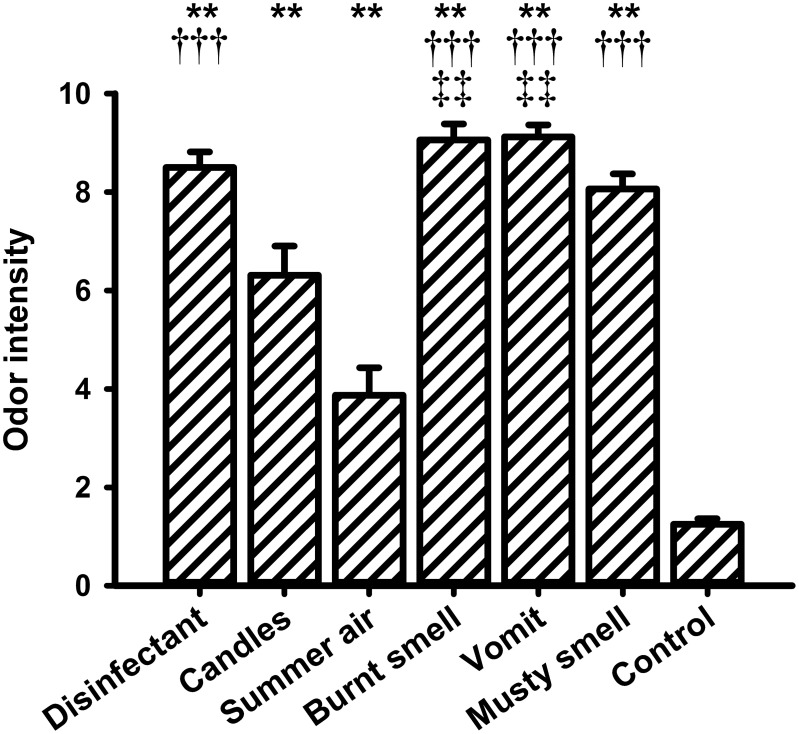
**Mean values (and s.e.m.) of the ratings of odor intensity**. ^**^Differs significantly (*P* < 0.01) from control, ^†††^differs significantly (*P* < 0.001) from “summer air,” ^‡‡^differs significantly (*P* < 0.01) from “candles.”

**Figure 7:**
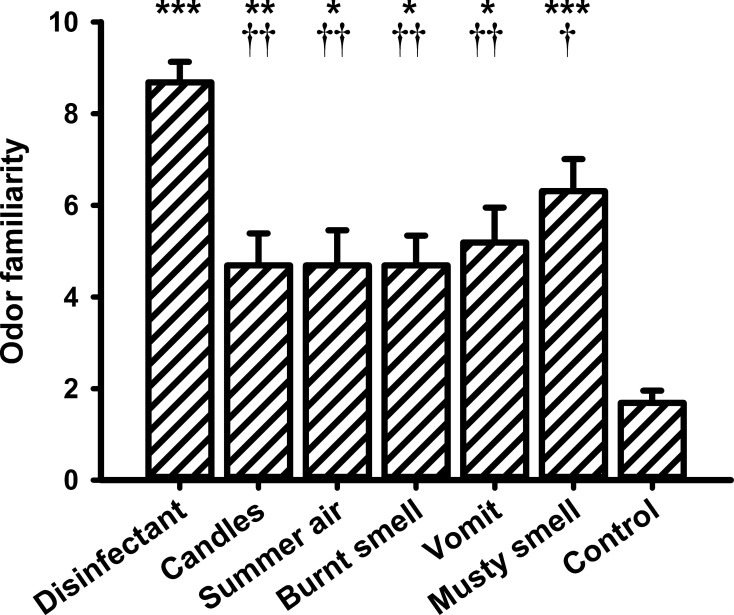
**Mean values (and s.e.m.) of the ratings of odor familiarity**. ^*^Differs significantly (*P* < 0.05) from control, ^**^differs significantly (*P* < 0.01) from control, ^***^differs significantly (*P* < 0.001) from control, ^†^differs significantly (*P* < 0.05) from “disinfectant,” ^††^differs significantly (*P* < 0.01) from “disinfectant.”

Figures 5–7 show the mean values of the valence, intensity, and familiarity ratings, respectively. With respect to the valence ratings, a One-Way repeated measures ANOVA revealed a significant main effect for the within-subjects factor “odor” [*F*_(6, 90)_ = 6.440, *P* < 0.001]. The highest valence rating was observed for “summer air,” whereas the lowest rating was recorded for “vomit.” *Post-hoc* pairwise comparisons revealed significant differences between “burnt smell” and “summer air” (*P* = 0.012), “burnt smell” and “musty smell” (*P* = 0.016) and “burnt smell” and odorless water (*P* = 0.019) as well as between “vomit” and “disinfectant” (*P* = 0.032), “vomit” and “summer air” (*P* = 0.004) and “vomit” and odorless water (*P* = 0.008).

For the intensity ratings the One-Way ANOVA showed a significant main effect for the within-subjects factor “odor” [*F*_(6, 90)_ = 66.308, *P* < 0.001]. The lowest intensity rating was observed for the control stimulus (i.e., odorless water). *Post-hoc* pairwise comparisons revealed significant differences between odorless water and all other fragrances (“disinfectant”: *P* = 0.000, “candles”: *P* = 0.000, “summer air”: *P* = 0.003, “burnt smell”: *P* = 0.000, “vomit”: *P* = 0.000, and “musty smell”: *P* = 0.000). “Summer air” also had a very low intensity rating and showed significant differences to “disinfectant” (*P* = 0.000), “burnt smell” (*P* = 0.000), “vomit” (*P* = 0.000), and “musty smell” (*P* = 0.000). “Summer air” further showed a marginally significant difference to “candles” (*P* = 0.051). The fragrance “candles” showed significant differences in intensity to “burnt smell” (*P* = 0.003) and “vomit” (*P* = 0.009).

With respect to the familiarity ratings the One-Way ANOVA also revealed a significant main effect for the within-subjects factor “odor” [*F*_(6, 90)_ = 13.627, *P* = 0.000]. The lowest familiarity rating was observed for the control stimulus (i.e., odorless water). *Post-hoc* pairwise comparisons showed significant differences between odorless water and all other fragrances (“disinfectant”: *P* = 0.000, “candles”: *P* = 0.007, “summer air”: *P* = 0.015, “burnt smell”: *P* = 0.014, “vomit”: *P* = 0.012, and “musty smell”: *P* = 0.000). “Disinfectant” received a very high familiarity rating and showed significant differences to “candles” (*P* = 0.009), “summer air” (*P* = 0.005), “burnt smell” (*P* = 0.003), and “vomit” (*P* = 0.014).

**Figure 8:**
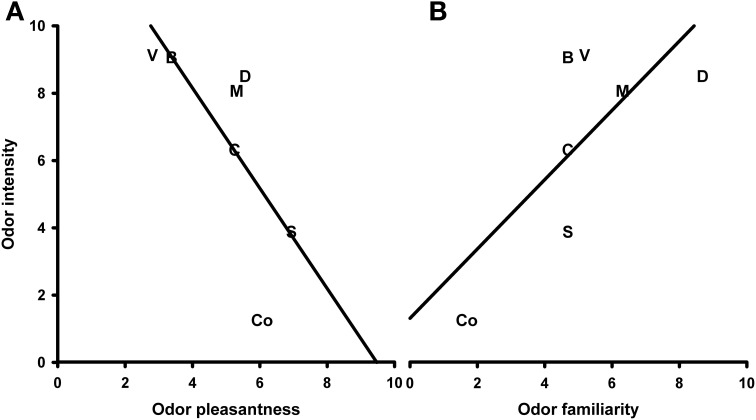
**Correlation between ratings of odor intensity and odor valence (A) and odor intensity and odor familiarity (B)**. S, Summer air; C, Candles; D, Disinfectant; B, Burnt smell; M, Musty smell; V, Vomit; Co, Control.

The correlation analysis showed a marginally significant negative correlation between the odor valence and intensity ratings (*N* = 7, *r* = −0.723, *P* = 0.067; Figure 8A). When this correlation was controlled for familiarity, it became highly significant (*N* = 7, *r* = −0.951, *P* = 0.004). Furthermore, a marginally significant, positive correlation between the intensity and familiarity ratings (*N* = 7, *r* = 0.719, *P* = 0.068; Figure 8B) was revealed. After controlling for the valence ratings, this correlation also became highly significant (*N* = 7, *r* = 0.950, *P* = 0.004). Finally, a partial positive correlation between the odor valence and familiarity ratings (controlled for intensity) was found (*N* = 7, *r* = 0.895, *P* = 0.016; uncontrolled *r* = −0.090, *P* = 0.847).

### Verbal labels

Table 2 shows the verbal descriptions of the olfactory stimuli given by the participants. In general, only about 25–50% of the subjects were able to put a name to the odors that were presented throughout the psychophysiological recordings even though the stimuli were presented again during the rating procedure. The only exception was “disinfectant” which was labeled by 24 out of 30 participants (80%). With respect to the labels, it is obvious that “disinfectant,” “burnt smell,” “musty smell,” “vomit” and the control odor were described quite accurately, whereas “summer air” and “candles” were never labeled correctly. However, in the case of “summer air” which was represented by the so called leaf alcohol the verbal labels demonstrate that subjects identified the “green” note of the fragrance that reminds of leaves and freshly cut grass.

**Table 2:** **Number of participants (*N* = 30) who named the olfactory stimuli and verbal labels (with number of nominations) for all odors**.

**Odor**	**No**.	**Verbal labels**
Summer air	14	Flowers/flower water/flowery meadow (8), conifer/fir tree (3), grass/grass clippings (3), lettuce (1), tomato (1)
Candles	8	Flowers/lilac/rose/cedar wood (6), banana (1), solvent (1)
Disinfectant	24	Disinfectant (13), alcohol/ethanol/isopropanol/solvent (11), doctor's office (2)
Burnt smell	13	Burnt smell/smoke/fire/lit match (6), wood/forest (4), leather (3), rubber (1), salami (1), dentist's office (1)
Musty smell	18	Soil/compost (8), musty/putrid/cellar (5), beet/carrot/asparagus/radish/red cabbage (6), flower (1), tires (1)
Vomit	12	Vomit/gastric acid/fermented/vinegar (5), peach/red currant/fruity (3), mold/organic waste (2), chewing gum (1), sweat (1), valerian (1)
Water (control)	7	Water (3), lotion with unobtrusive smell (1), no odor (1), paper (1), rose (1)

### Cognitive association between olfactory stimuli and basic emotions

The χ^2^ test revealed that the odors “candles” (χ^2^ = 31.6, *P* = 0.000) and “summer air” (χ^2^ = 17.2, *P* = 0.001) were both associated specifically with the basic emotion “happiness,” whereas “vomit” (χ^2^ = 33.2, *P* = 0.000) and “burnt smell” (χ^2^ = 12.0, *P* = 0.017) were both associated specifically with the basic emotion “disgust.” The odors “disinfectant” und “musty smell” were not specifically related to a single basic emotion (*P* > 0.1). It is important to note that the control stimulus (i.e., odorless water) was specifically associated with no basic emotion (χ^2^ = 62.8, *P* = 0.000). Thus, four odors could be associated with a single basic emotion in this study, but only two of these odors (“vomit” and “summer air”) could be associated with the hypothetical basic emotion (see Table 3).

**Table 3:** **Association (number of nominations) of the olfactory stimuli with the basic emotions**.

**Odor**	**Hap**	**Sur**	**Fea**	**Ang**	**Sad**	**Dis**	**Uns**
Summer air	16^*^	4	0	0	0	1	9
Candles	16^*^	**3**	1	0	1	5	4
Disinfectant	8	3	**3**	1	3	6	6
Burnt smell	3	7	3	**0**	0	13^*^	4
Musty smell	7	7	0	0	**0**	11	5
Vomit	1	4	0	0	0	21^*^	4
Water (Control)	4	1	0	1	1	2	21^*^

*Numbers in bold indicate a match between the hypothetical and the observed association. Hap, happiness; Sur, surprise; Fea, fear; Ang, anger; Sad, sadness; Dis, disgust; Uns, no or unspecific association*.

* *Indicates that the verbal association was emotion-specific*.

## Discussion

In the present study, we aimed to evaluate the emotional potency and distinctiveness of six odors that were connoted with the olfactory environment of the City of Vienna. Based on earlier reports on the induction of discrete emotions by odors (e.g., Alaoui-Ismaili et al., 1997a; Robin et al., 1999; Vernet-Maury et al., 1999) we hypothesized that urban odors elicit emotional responses that can be distinguished by physiological activity. Since the study of Robin et al. (1999) showed that the emotional response toward an odor is shaped by prior subjective experience, we sought to account for this finding when selecting the odors for the current investigation by taking into account autobiographical factors.

### Autonomic nervous system parameters and subjective odor ratings

Our data did not show any emotion specific autonomic response patterns as a result of the olfactory stimulation. Although the parameters chosen in this study resemble those in the investigation of Ekman et al. (1983) and have also been used by others to detect emotion-specific autonomic activity in response to sensory stimuli (for details see Kreibig, 2010), our array of parameters differs from that of Alaoui-Ismaili et al. (1997a) in their olfactory studies. Thus, we may have failed to choose the appropriate set of physiological endpoints to detect olfactory induced emotions. This seems plausible as a recent investigation (Croy et al., 2013) demonstrated that systolic blood pressure responses differed depending on the sensory channel used to induce disgust. With regard to individual autonomic parameters, we found that the amplitude of the SCR varied as a function of odor presentation. In addition, hedonic odor valence was negatively correlated with the amplitude of the SCR. Thus, our results indicate that electrodermal activity differentiates between pleasant and unpleasant odors. These observations are in line with previous findings of Delplanque et al. (2009) but in contrast with the findings of Moller and Dijksterhuis (2003), who found no evidence for a relationship between odor pleasantness and the amplitude of the SCR using four iso-intense non-trigeminal odors. Bensafi et al. (2002a) reported a marginal correlation between electrodermal activity and odor intensity which was also revealed in our study. The magnitude of the electrodermal response is believed to reflect the activation level of the sympathetic branch of the ANS (Critchley, 2002; Sequeira et al., 2009). Since hedonic odor valence and odor intensity ratings were strongly correlated in our study, we cannot fully rule out the possibility that the effect on electrodermal activity was driven by odor intensity or potential differences in the trigeminal activity of the odors.

In regard to cardiovascular activity, we found that HRV decreased as the fragrances were rated more intense and less pleasant. Similar relationships between heart rate variations and subjective ratings of odor pleasantness have been described by Bensafi et al. (2002a). Aue and Scherer (2008) reported smaller changes in heart rate in response to unpleasant as opposed to pleasant pictures. HRV has been linked with regulated emotional responding, and reduced overall, and parasympathetically mediated HRV has been observed in several forms of anxiety and depression (Appelhans and Luecken, 2006). Thus, our results could probably be interpreted in terms of diminished regulated emotional responding accompanying negative emotional states such as fear and sadness as the olfactory stimuli were perceived as more intense and less pleasant. An alternative explanation is that odors which were rated high in intensity and low in pleasantness induced sympathetic activation (Inoue et al., 2003) resulting in reduced HRV.

With respect to the ratings of perceived odor pleasantness, intensity, and familiarity, the results of the present study showed that the unpleasant odors “vomit” and “burnt smell” differed significantly from the pleasant fragrance “summer air” and from the control odor. Regarding intensity, all odors differed significantly from the weak odor “summer air” and from the control odor. Finally, “candles” rated intermediate in intensity differed from the very strong odors “vomit” and “burnt smell.” The analyses of the familiarity ratings showed that both the most familiar odor, i.e., “disinfectant,” as well as the least familiar control odor differed significantly from all other odors. The correlation between the change in ST and the odor familiarity ratings indicated that ST decreased with increasing odor familiarity. To the best of our knowledge such a relationship has never been observed before and more research is needed to interpret this finding.

### Verbal labels and cognitive associations

The analyses of the verbal responses showed that only 25–50% of the participants could produce a label for the presented odors. In addition, some odors were harder to name than others. In particular, verbal descriptions for “disinfectant,” “burnt smell,” “musty smell,” “vomit,” and the control odor were accurate in most cases, whereas none of the subjects used the labels “summer air” and “candles” for the respective fragrances. Difficulties in odor naming are a common finding (Cain, 1979) and are particularly relevant in verbal odor identification tasks. To account for this general deficit odor identification is often facilitated in such tasks by offering a number of alternatives from which the correct label must be chosen. As we were interested in free associations rather than correct identification in this study we decided against the use of verbal cues. The odor naming deficit is often observed even for very familiar odors (Olofsson et al., 2013). In the present study, however, the number of label use seemed to go hand in hand with the familiarity ratings. “Disinfectant” which received the highest familiarity rating was named by 80% of the participants and was followed in terms of labeling by several odors with similar familiarity ratings. The control odor which was rated least familiar also had the lowest count of labels used.

In regard to the verbal associations between the odors and the basic emotions it is obvious that fragrances with high pleasantness and low intensity ratings were associated with happiness, whereas those with low pleasantness and high intensity ratings were associated with disgust. Similar observations have also been made in the visual (Barrett and Niedenthal, 2004) and in the olfactory domain (Alaoui-Ismaili et al., 1997a; Robin et al., 1999; Weber and Heuberger, 2011). Levenson stated that when sensory stimuli are used for emotion induction subjects “report feeling emotions that […] represent their judgments of the emotional qualities of the stimuli” rather than experiencing emotions (Levenson, 2003, p. 217). However, with the current experimental paradigm we can neither confirm nor reject this argument. We found that only four out of six odors, i.e., “summer air,” “candles,” “burnt smell,” and “vomit” were uniquely assigned to a single basic emotion. Moreover, only two of the six emotions, i.e., happiness and disgust, were specifically associated with an odor. The latter finding is in line with previous observations on the relationship between basic emotions and verbal associations (Alaoui-Ismaili et al., 1997a; Bensafi et al., 2002b; Croy et al., 2011) and can be explained by the results of Chrea et al. (2009) and Delplanque et al. (2012). Nevertheless, the practical constraints in the odor selection process that have been outlined in the Introduction may also have contributed to these results.

In conclusion, our results suggest that urban odors may evoke specific cognitive concepts of basic emotions. Moreover, both autonomic activity and cognitive associations elicited by such odors seem to be related to odor hedonics and odor strength without being necessarily emotion specific. Our findings might be relevant in the field of urban design in that they underscore the emotional potency of odors connoted with an urban environment while at the same time they discourage ambitions to deliberately induce specific affective states utilizing ambient odors in public spaces.

## Author contributions

Sandra T. Glass and Eva Heuberger conceived and designed the experiments, Sandra T. Glass and Elisabeth Lingg collected and analyzed the data, Sandra T. Glass and Eva Heuberger wrote the paper.

### Conflict of interest statement

The authors declare that the research was conducted in the absence of any commercial or financial relationships that could be construed as a potential conflict of interest.
