# Supplementary material for: Do ambient urban odors evoke basic emotions?
Source: Front Psychol. 2014 Apr 23;5:340. doi: 10.3389/fpsyg.2014.00340 (PMC4017720; doi:10.3389/fpsyg.2014.00340)
Supplement: Supplementary file 1 [file Presentation1.PDF]

Supplementary material

Chemicals

Ethanol 96% (Ph. Eur.) and hydrochloric acid (36%) were obtained from Phönix Arzneiwarengroßhandlung Ges.m.b.H, Austria. Propan-1,2-diol puriss. p.a. ACS, ≥ 99.5 % (GC), 2-methoxyphenol purum ≥ 98% (GC) and 3-methylbutanoic acid puriss. ≥ 98.5% (GC) were purchased from Fluka (Sigma Aldrich, Germany). propan-2-ol, (4R,4aR,8aS)-4,8a-dimethyl-1,2,3,4,5,6,7,8-octahydronaphthalen-4a-ol ≥ 98 % (~ 2 mg/ml in methanol) and butanoic acid ≥ 98 % were obtained from Sigma Aldrich, Austria. 2-Methoxy-3,5 or 6-(propan-2-yl)pyrazine ≥ 98% and Z-hex-3-en-1-ol ≥ 98% natural (FCC, FG) were products of SAFC (Sigma Aldrich, Austria). Methyl 2-phenylacetate was obtained from Kurt Kitzing GmbH, Germany.

Table S1: Mean values (Mean) and SEM of the latency and half recovery time of the skin conductance response (SCR), the changes of heart rate (HR Dif), heart rate variability (HRV Dif), number of breaths (BR Dif), number of eye-blinks (EBR Dif), forearm muscle activity (EMG Dif), and skin temperature (ST Dif) in response to the olfactory stimuli and the control.

	**SCR latency**		**SCR half recovery time**		**HR Dif**	
	Mean	*SEM*	*Mean*	*SEM*	*Mean*	*SEM*
Summer air	2.308	0.135	1.617	0.236	-2.999	0.620
Candles	2.372	0.099	1.365	0.127	-3.408	0.554
Disinfectant	2.638	0.115	1.416	0.115	-2.855	0.528
Burnt smell	2.603	0.109	1.339	0.088	-3.224	0.520
Musty smell	2.474	0.116	1.509	0.175	-3.363	0.650
Vomit	2.501	0.097	1.520	0.096	-2.683	0.562
Water (control)	2.597	0.153	1.245	0.084	-2.618	0.425

	**HRV Dif**		**BR Dif**		**EBR Dif**	
	Mean	*SEM*	*Mean*	*SEM*	*Mean*	*SEM*
Summer air	0.653	0.313	0.047	0.171	-0.109	0.395
Candles	0.348	0.402	0.000	0.194	-0.047	0.423
Disinfectant	0.674	0.443	0.094	0.211	-0.016	0.358
Burnt smell	0.109	0.470	0.172	0.182	0.094	0.416
Musty smell	0.089	0.434	0.109	0.188	-0.016	0.470
Vomit	-0.274	0.497	0.141	0.187	-0.292	0.389
Water (control)	0.884	0.598	-0.094	0.208	-0.203	0.447

	**EMG Dif**		**ST Dif**	
	Mean	*SEM*	*Mean*	*SEM*
Summer air	0.053	0.053	-0.000	0.004
Candles	0.036	0.036	-0.002	0.002
Disinfectant	0.028	0.028	-0.006	0.004
Burnt smell	0.010	0.010	0.009	0.003
Musty smell	-0.036	-0.036	-0.003	0.004
Vomit	0.035	0.035	0.000	0.003
Water (control)	-0.012	-0.012	0.005	0.003
